# The Effect of Albumin/Glutaraldehyde Glue (Bioglue) on Colonic Anastomosis Under Intestinal Obstruction: An Experimental Study in Rats

**DOI:** 10.3390/jcm14072457

**Published:** 2025-04-03

**Authors:** Kalliopi Despoudi, Ioannis Mantzoros, Orestis Ioannidis, Elissavet Anestiadou, Savvas Symeonidis, Stefanos Bitsianis, Efstathios Kotidis, Manousos George Pramateftakis, Antonia Aikaterini Bourtzinakou, Eleni Salta-Poupnara, Konstantinos Angelopoulos, Barbara Driagka, Freiderikos Tserkezidis, Stamatios Angelopoulos

**Affiliations:** 4th Department of Surgery, General Hospital “George Papanikolaou”, Aristotle University of Thessaloniki, 57010 Thessaloniki, Greece; kdesp@gmail.com (K.D.); imanvol@gmail.com (I.M.); elissavetxatz@gmail.com (E.A.); simeonidissavvas@yahoo.com (S.S.); sbitsiani@gmail.com (S.B.); skotidis@gmail.com (E.K.); mpramateftakis@hotmail.com (M.G.P.); katerbourtzi@gmail.com (A.A.B.); elenisalta98@gmail.com (E.S.-P.); kostaggelo@hotmail.com (K.A.); valiadrg@gmail.com (B.D.); frtserkezidis@gmail.com (F.T.); saggelopoulos@auth.gr (S.A.)

**Keywords:** bursting pressure, Bioglue albumin/glutaraldehyde, collagen, colonic anastomosis, obstructive ileus, anastomotic leak

## Abstract

**Background/Objectives**: Healing of colonic anastomoses is critical to surgical recovery, particularly under obstructive ileus conditions. Adhesive biological materials such as albumin/glutaraldehyde glue (Bioglue) show potential in enhancing anastomotic healing and minimizing complications. This study investigates the effect of Bioglue on colonic anastomoses healing under obstructive ileus conditions in rats. **Methods**: Eighty albino Wistar rats were divided into control, ileus, Bioglue, and ileus + Bioglue groups (*n* = 20 each). Subgroups (*n* = 10) were sacrificed on the 4th or 8th postoperative day. In the control and Bioglue groups, end-to-end anastomoses were performed after colonic resection. In the ileus and ileus + Bioglue groups, obstructive ileus was induced by colonic ligation, followed by resection and primary anastomosis. Bioglue was applied in the Bioglue and ileus + Bioglue groups. Assessments included bursting pressure, peritoneal adhesion and inflammation scores, and biochemical markers (fibroblast activity, neoangiogenesis, collagen deposition, hydroxyproline, and collagenase concentrations). **Results**: Bursting pressure and fibroblast activity were significantly higher in the ileus + Bioglue group compared to the ileus group on both postoperative days. Although anastomotic rupture occurred in the ileus and ileus + Bioglue groups, the incidence was not significantly different from the control and Bioglue groups. Ileus + Bioglue showed significantly higher adhesion scores, inflammatory infiltration, neoangiogenesis, and collagen deposition compared to the control and ileus groups. Hydroxyproline was significantly elevated in the ileus + Bioglue group on the 8th day. Collagenase I concentrations were higher in ileus + Bioglue but not significant. **Conclusions**: Bioglue application enhances colonic anastomotic healing under obstructive ileus conditions, improving mechanical strength and promoting tissue repair by the 4th and 8th postoperative days. These findings support its potential clinical application.

## 1. Introduction

Anastomotic dehiscence is a serious complication after colorectal surgery, as it increases postoperative morbidity and mortality rates, and also negatively affects healthcare costs and oncologic outcomes, especially local recurrence rates and failure to receive adjuvant chemotherapy [1,2]. Its incidence ranges between 2% and 19%, and differences among authors may be attributed to a lack of consensus regarding the definition of anastomotic leakage [3]. However, anastomotic rate may rise to 25% of the cases in the presence of an underlying condition, such as malignancy or previous surgery combined with intraperitoneal hyperthermic chemotherapy [4]. Anastomotic dehiscence can present either as a rupture or as a simple leakage. Rupture most often occurs between the 2nd and the 5th postoperative days, and leakage may present until the 7th postoperative day [5]. The literature contains numerous classifications of anastomotic leak grade, including the classification proposed by the International Study Group of Rectal Cancer, which is based on clinical management modalities (grade A, no change in management; grade B, active therapeutic intervention without the need for re-laparotomy; grade C, need for re-laparotomy) and by Lim and colleagues, who classified anastomotic leakage according to presentation: generalized peritonitis (type I), localized peritonitis (type II), and fistula or chronic sinus (type III) [6,7].

Furthermore, anastomotic leakage complicating colorectal procedures constitutes a major economic burden for healthcare systems. Kang et al., in their retrospective, nationwide analysis, conclude that anastomotic leakage led to increased mean costs for the index hospitalization, increased postoperative costs, readmission rates and costs, as well as longer mean length of stay during readmissions, when patients develop complications due to anastomotic leakage were compared to patients with intact anastomosis postoperatively [8]. The factors that influence the healing of anastomosis can be divided into two major categories, systemic (age, anemia, nutritional status, medications, hypoalbuminemia, more than one associated comorbidity, diabetes mellitus, cirrhosis, blood transfusion, preoperative radiation, anastomosis conducted in emergency setting, colonic anastomosis, preoperative leukocytosis) and local (blood supply, intraoperative contamination, surgical technique, bowel preparation, perioperative chemoprophylaxis, peritonitis and ileus) [9,10]. The creation of protective ileostomy or colostomy more centrally of the anastomosis not only does not appear to affect healing, but has also been associated with increased incidence of complications such as disuse enteritis and intestinal obstruction [11].

Postoperative ileus (PPOI) is common after colorectal surgery, both open and laparoscopic, with important effects on postoperative course, mortality, and readmission rates [12]. The literature data suggest that the incidence of PPOI following colorectal procedures ranges from 2% to 54% [13]. In recent years, ileus has been associated with numerous intraabdominal complications, and especially anastomotic dehiscence. However, up to date, the direction of the link between ileus and anastomotic leakage remains obscure [14].

Numerous background pathophysiological mechanisms have associated ileus with improper healing of the anastomosis after colon resection. The dilated central part of the intestine, leading to impaired blood supply in its wall, as well as the significant difference in the diameter of the two anastomotic segments can lead to an increased rate of anastomotic dehiscence. Additionally, increased intraabdominal pressure due to small bowel and colon distension can also result in reduced blood flow, pressure, and ischemia in the anastomosis [14]. Furthermore, hypovolemia and electrolytic disorders caused by ileus are responsible for inhibiting healing [15]. Based on experimental findings, Peters and colleagues reported the important role of systemic inflammation in the relationship between anastomotic leak and PPOI [16]. In conclusion, intestinal obstruction negatively impacts anastomotic healing by impairing blood supply, increasing intraluminal pressure, and inducing ischemia-reperfusion injury, which collectively disrupt normal tissue repair.

The seriousness and economic burden of the anastomotic dehiscence has aroused researchers’ interest, and in recent years, there have been several ongoing experimental studies aimed at finding more effective methods for protecting the anastomosis. Despite scientific research and the investigation of protective materials, the ideal protective agent to reduce anastomotic dehiscence rate has not been established yet [4].

Various adhesive biological materials have been evaluated for their potential to enhance anastomosis (fibrin adhesive, fibrin sealant, fibrin glue, Floseal, cyanoacrylate glue) [17,18,19,20]. These adhesives are biodegradable and biocompatible and have been used in numerous procedures [21]. However, existing options, such as fibrin glue and cyanoacrylate glue have notable limitations that impact their efficacy in anastomotic reinforcement. Fibrin glue, despite its biocompatibility and ease of application, has limited mechanical strength and rapid degradation, reducing its ability to provide long-term structural support [22]. Additionally, its reliance on exogenous thrombin raises concerns about immune reactions and variable clot stability in patients with coagulopathies [23]. On the other hand, cyanoacrylate-based adhesives offer strong bonding capabilities but suffer from high tissue toxicity, leading to local inflammation, necrosis, and a potential foreign body reaction [24]. These drawbacks necessitate the exploration of alternative bioadhesives with enhanced mechanical strength, biocompatibility, and controlled degradation to optimize anastomotic healing. However, concerns regarding long-term biocompatibility and potential inflammatory responses remain, warranting further investigation into its safety and efficacy in colorectal anastomoses.

In 1998, albumin/glutaraldehyde (Bioglue) tissue sealant, an FDA-approved dual-composition adhesive consisting of 45% of purified bovine albumin (BSA) solution and 10% of glutaraldehyde solution (4:1 ratio), was first used in cardiac surgery [25,26,27]. Later on, applications of Bioglue also extended to a wider spectrum, in addition to established methods of surgical repair, in order to strengthen soft tissues, such as pulmonary, vascular, and urogenital [28,29,30,31,32]. Bioglue addresses some of the abovementioned challenges by providing superior tensile strength and prolonged tissue adhesion.

However, the literature contains limited reports regarding the effect of Bioglue on healing anastomosis after colorectal procedures, mainly in rat experimental models [33]. Furthermore, Ekici and colleagues were the first to investigate the role of Bioglue in the healing of normal and impaired colonic anastomosis [34]. Despoudi et al. proved that obstructive ileus is an important factor for high-risk anastomosis [35], as well as the protective role of Bioglue on colonic anastomosis healing [36]. Regarding clinical trials, a pilot study by de la Portilla et al. evaluated its use in the treatment of high transsphincter anal fistulas, demonstrating a 50% success rate in fistula healing after a mean follow-up of 13.92 months [37]. While these preliminary results suggest potential utility, this study highlights the need for further long-term prospective trials to establish its efficacy and safety in colorectal applications. No other large-scale clinical trials have been conducted to evaluate Bioglue’s efficacy and long-term safety in human colorectal anastomoses. Given its promising mechanical properties, further research is warranted to assess its clinical applicability, biocompatibility, and potential risks in colorectal surgery.

As a result, our team designed the present experimental study to investigate the effect of local application of Bioglue on the healing of colonic anastomosis under obstructive ileus conditions in rats and to explore if Bioglue can reverse the negative effects of ileus in anastomotic integrity. We hypothesize that the local application of Bioglue (CryoLife, Atlanta, GA, USA) on colonic anastomoses under obstructive ileus conditions enhances anastomotic healing by improving mechanical strength, promoting tissue repair, and mitigating the adverse effects of ileus-induced ischemia. This study aims to assess the impact of Bioglue on anastomotic integrity through histopathological and biochemical analyses, evaluating its potential role in reducing anastomotic dehiscence and improving healing outcomes.

## 2. Materials and Methods

### 2.1. Experimental Animals

This study was a prospective, randomized, double-blind experimental study. All necessary approved protocols for laboratory animal care were ensured. The experiment was performed, and the results were published in accordance with the ARRIVE guidelines 2.0 [38].

Eighty male rats of the Wistar species, 3–4 months old, each weighing 200–300 g, were used for this study. Male rats were chosen to avoid the confounding effects of hormonal fluctuations associated with the estrous cycle, which can influence wound healing and immune response. An experimental animal protocol was designed to minimize pain and discomfort in the animals. The rats were under a 12 h cycle of light and dark conditions for seven to ten days prior to the surgery. Each animal was individually housed and had unlimited access to both water and food before and after the surgery. No antibiotics were administrated. The sacrifice was performed using intracardial infusion of 10% KCL solution.

Both the maintenance of the rats and the experimental procedures were conducted at the Experimental Laboratory of the Veterinary School of A.U.Th. under the supervision of a veterinarian responsible for ensuring the hygiene of the facilities and maintaining the appropriate conditions for the protection of the laboratory animals. This experimental study was approved by the Ethical Committee of the Department of Veterinary Services of the Prefecture of Thessaloniki (S.N.: 13/10767/15 September 2003). The experiments were performed in accordance with the provisions of the European Convention for the protection of animals used for experimental and research purposes (Law 1197/81 Art. 4, Law 2015/92, PD 160/91). This study was conducted under the supervision and with the contribution of a veterinarian, who was responsible for ensuring compliance with hygiene regulations in the premises, as well as the rules of acclimatization, protection, and nutrition of the experimental animals.

### 2.2. Study Groups

Eighty male rats were randomly divided into 4 groups of 20 rats each and each of them into 2 subgroups depending on the day of sacrifice, leading to 8 subgroups of 10, using electronic randomization software. Each animal corresponded to a unique three-digit number. The randomization process was conducted using Research Randomizer software (Version 4.0) by Urbaniak & Plous.

Control group (*n* = 20 animals): a 1 cm segmental colonic resection was performed 5 cm cranial to the rectum, followed by a primary end-to-end anastomosis.

Control1 (10 animals): the sacrifice took place on the 4th postoperative day.
Control2 (10 animals): the sacrifice took place on the 8th postoperative day.

Ileus group (*n* = 20 animals): the left colon was ligated at 5 cm cranial to the rectum using a silk 3/0 suture. After 24 h, a colonic resection of the obstructed part 1 cm around the ligation and a primary end-to-end anastomosis were performed.

Ileus1 (10 animals): the sacrifice took place on the 4th postoperative day.
Ileus2 (10 animals): the sacrifice took place on the 8th postoperative day.

Bioglue group (*n* = 20 animals): a 1 cm segmental colonic resection was performed 5 cm cranial to the rectum, followed by a primary end-to-end anastomosis. After creating the anastomosis, albumin/glutaraldehyde sealant [Bioglue^®^ (CryoLife, Atlanta, GA, USA)] was locally applied around it.

Bioglue1 (10 animals): the sacrifice took place on the 4th postoperative day.
Bioglue2 (10 animals): the sacrifice took place on the 8th postoperative day.

Ileus + Bioglue group (*n* = 20 animals): the left colon was ligated at 5 cm cranial to the rectum using a silk 3/0 suture. After 24 h, a colonic resection of the obstructed part, 1 cm around the ligation, and a primary end-to-end anastomosis were performed, and albumin/glutaraldehyde was locally applied around it.

Ileus + Bioglue1 (10 animals): the sacrifice took place on the 4th postoperative day.
Ileus + Bioglue2 (10 animals): the sacrifice took place on the 8th postoperative day.

Our ileus induction technique aligns with previously published methods in experimental models of bowel obstruction, since the use of bowel ligation, with either a silicon ring or sutures is a widely used and feasible method to model conditions of bowel obstruction [39,40].

### 2.3. Anesthesia and Operative Technique

Surgery was performed through a 3 cm midline laparotomy. A total of 40 mg/kg of thiopental was administered intraperitoneally for anesthesia. Afterwards, a 1 cm segmental colonic resection was performed in both the control and Bioglue groups, followed by a primary end-to-end anastomosis. Primary end-to-end anastomosis refers to the direct surgical reconnection of the ends of two segments of the colon following resection, without the creation of a protective stoma. The proximal and distal colonic parts were manually decompressed from fecal content prior to anastomosis. In the ileus and ileus + Bioglue groups, the left colon was ligated at 5 cm cranial to the rectum for ileus establishment using a 3/0 silk suture. After 24 h, a colonic resection of the obstructed part 1 cm around the ligation and a primary end-to-end anastomosis were performed. In all rats, a single layer of eight extramucosal interrupted 6-0 poly-propylene sutures were used for the anastomosis in a single layer fashion, including individual sutures placed in the seromuscular layer of the intestinal wall without penetrating the mucosa. In this study, we selected single interrupted polypropylene sutures, as they allow for precise tension control and uniform placement, minimizing variability in experimental conditions. While continuous absorbable sutures are commonly used in clinical intestinal anastomoses, polypropylene sutures are widely utilized in experimental models due to their non-degradable nature, ensuring stability throughout the study period. This choice facilitates the accurate assessment of anastomotic healing without the confounding effects of early suture absorption. Additionally, previous experimental studies investigating anastomotic reinforcement strategies have successfully employed polypropylene sutures, making them a well-established option in this setting [41,42]. In this study, a Lembert suture pattern was used to ensure a secure, inverting closure. Additionally, in our experiment, a single-layer extramucosal interrupted suture technique was used, as it has been widely validated in experimental settings for its reproducibility and minimal confounding variables [43,44]. In the Bioglue and ileus + Bioglue groups, albumin/glutaraldehyde glue was also applied around the anastomosis with an applicator, taking care to avoid dispersion into the peritoneal cavity. The peritoneal cavity was irrigated, and the abdominal wall was closed using 3-0 silk sutures.

**Figure 1:**
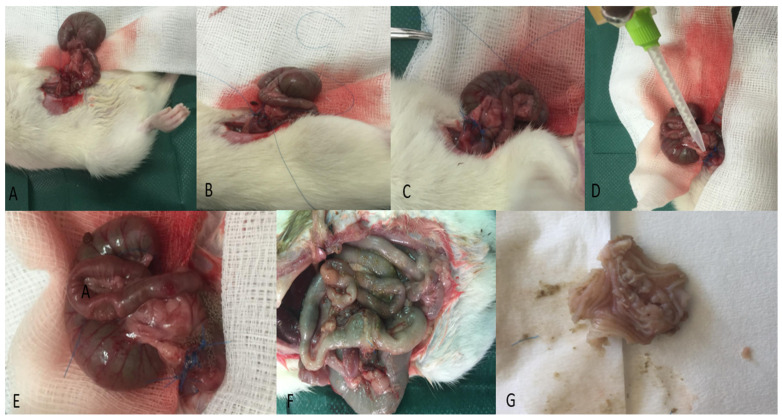
An overview of the experimental procedure: (**A**) stay suture marking the beginning of the anastomosis; (**B**) one side of the hand-sewn anastomosis; (**C**) completed colonic anastomosis in a single layer with eight extramucosal interrupted 6-0 polypropylene sutures; (**D**) topical application of Bioglue at the anastomotic site; (**E**) reinforcement of anastomosis with Bioglue; (**F**) anastomotic leak in a rat from the ileus + Bioglue group; (**G**) 2.5 cm colon segment encompassing the anastomosis.

After the operation, the experimental animals were kept in pairs in cages, where they were monitored for an hour until they fully recovered and regained normal mobility. All experimental animals had free access to food and water from the day of surgery until sacrifice. An overview of the experimental procedure is shown in Figure 1.

### 2.4. Body Weight Measurement

All animals’ body weight was measured at the beginning of the experiment and immediately prior to sacrifice. Measurements were performed by using precision scales, according to a code assigned to each animal after the anesthesia was administered.

### 2.5. Macroscopic Examination

On the sacrifice day, the animals underwent midline laparotomy under proper anesthesia. During the operation, the anastomotic parts of the intestine were resected and examined macroscopically. The presence of peritonitis, adhesions, or abscess near the anastomosis, as well as anastomotic integrity, were evaluated using the van der Ham scale [45], in which a score of 0 represents absence of adhesion formation, a score of 1 a minimum number of adhesions, a score of 2 moderate adhesions between the anastomosis and, for example, an intestine loop, and a score of 3 represents severe adhesion formation plus the presence of abscess.

### 2.6. Bursting Pressure Measurement

The bursting pressure of anastomosis was evaluated ex vivo. A 2.5 cm segment of the colon containing the anastomosis was removed along with the attached adhesions. This was followed by the thorough cleansing of the intestine to remove fecal content and meticulous rinsing of the specimen with normal saline. The proximal segment of the removed colon was closed with a silk 3-0 suture, and then a three-way catheter was inserted into the distal part. The catheter was secured at the bursting pressure measuring device, which consisted of a custom-made setup incorporating a basic manometer, a tube attached to the three-way cannula, and a syringe filled with dyed water connected to the three-way cannula. The specimen was secured to the tube with a purse-string suture, while its free edge was clamped. The catheter was fixed to the bursting pressure apparatus, as described in previous research [46,47]. Through this, the lumen of the bowel was filled with normal saline solution at a 1ml/min rate. The pressure at which a leakage of normal saline or rupture occurred, measured in mmHg, was defined as the bursting pressure. The exact site of leakage or rupture during the measurement of bursting pressure was noted, due to the fact that in some rats that point was noted at the site of the anastomosis, while in others, it was noted at another point.

### 2.7. Histopathological Assessment

After the bursting pressure was measured, the mesentery and fat were removed from the resected segment of the intestine, which was then cleaned with normal saline. The part of the colon containing the anastomosis was resected, with margins of 0.5 cm on each side, and then divided into two parts at a level perpendicular to the longitudinal axis of the intestine to ensure precise anatomical orientation. One part of the segmented colon was placed in a 4% formaldehyde solution for histology examination and then stained with eosin-haematoxylin stain. The anastomosis was evaluated histologically, under a light microscope, using the 0–4 Erlich–Hunt grading scale with the Phillips et al. modification [48]. The parameters evaluated included the new blood vessels formation, the infiltration of inflammatory cells, the activity of fibroblasts, and the collagen deposition [49]. These parameters were evaluated individually using a numbered scale: 0 (-) = no evidence; 1 (+) = occasional evidence; 2 (++) = light scattering; 3 (+++) = abundant evidence; and 4 (++++) = confluent fibers or cells.

### 2.8. Hydroxyproline Measurement

The collagen deposition in the anastomosis was assessed by evaluating the hydroxyproline percentage [50]. For that purpose, the second segment of the resected intestine with the anastomosis was weighed and kept at −20 °C after being divided vertically into two segments. The hydroxyproline quantity in the tissue was measured with modified experimental methods, as described be Reddy et al. and Hewitt et al. [28,51]. At first, the samples were lyophilized, then the tissue specimens were homogenized with the use of a polytron homogenizer in distilled water. The collagen that was soluble in acid was removed from the tissue of the specimens using incubation overnight with an acetic acid solution of 0.5 mol/L at 4 °C. An amount of 70 μL of standard/test sample was hydrolyzed in 30 μL NaOH 10.125 mol/L for 25 min at 120 °C by autoclaving. After hydrolyzation, the sample was blended with a chloramines-T reagent buffer (0.056 mol/L) at room temperature. The oxidation process lasted for 25 min. Chromophore growth was monitored by adding Ehrlich’s reagent. The absorption was evaluated at 550 nm using a Biotek μQuantTM spectrophotometer. The presence of hydroxyproline in unknown tissue extracts was determined from the standard curve by plotting absorbance values against the concentration of standard hydroxyproline. The outcomes were evaluated in μg per gram of tissue [51].

### 2.9. Type I Collagenase Measurement

Type I collagenase density was also measured in another segment of the anastomotic area using an ELISA kit (USCNLIFEm E0212r). A plate was precoated with specific antibodies for type I collagenase (polyclonal, conjugated to biotin). Then, the control sample was added. Avidin, which was also conjugated to hydroxyproline, was also added to the plate and hatched. The development of chromophore occurred after adding TMB substrate solution. A sulfuric acid solution was used in order to terminate the reaction. The alteration in color was evaluated at 450 nm, using spectrophotometry (by Stat Fax-210TM spectrophotometer (Awareness Technology Inc., Palm City, FL, USA). Type I collagenase density in random tissue specimens was extracted from the standard curve. The results were measured in μg per gram of wet tissue [51].

### 2.10. Statistical Analysis

The data obtained from the experiment were summarized using statistical descriptive indices of absolute and relative frequencies (percentages %), indices of central tendency (means, medians, 95% confidence intervals), and dispersion indices (minimum and maximum values, standard deviations). For each parameter, the Least Significant Difference test (LSD) was used to compare mean values. The statistical analysis of results was performed using the ANOVA (Analysis of Variance) test and Fisher’s Exact Test for comparison of percentages (%). The level of statistical significance was pre-set at a *p* value < 0.05 for the comparisons between groups and at *p* < 0.10 for percentage comparisons. The latter was selected due to the relatively small sample size, which may lead to underpowered results when assessing categorical differences. While *p* < 0.1 does not indicate definitive statistical significance, it allows us to identify potential trends that may warrant further investigation in future studies. IBM SPSS Statistics for Windows, Version 22.0 (IBM Corp., Armonk, NY, USA) enhanced with the module Exact Tests was used for all the statistical analyses. Bar charts were designed for the diagrammatic presentation of the results.

## 3. Results

No deaths or wound infections occurred, and no anastomotic leak was noted before sacrifice day. The results concerning the comparison between the control and the Bioglue groups [36], as well as that between the control and ileus groups [35] have already been published by authors, and they will not be further discussed in this paper.

### 3.1. Body Weight

The mean body weight was calculated both preoperatively and on the day of sacrifice (4th and 8th postoperative days). The difference between the mean values was evaluated (Table S1, Supplementary Materials). According to the statistical analysis, the mean weight between the subgroups did not differ significantly at the beginning of the experiment (*p* = 0.707). However, the mean body weight differed between the groups at the end of the experiment.

On the 4th postoperative day, the mean body weight reduction in the ileus + Bioglue1 subgroup was significantly lower compared to the ileus1 subgroup (*p* < 0.001), indicating that Bioglue mitigated weight loss under obstructive ileus conditions. Compared to the control1 and Bioglue1 subgroups, the ileus + Bioglue1 subgroup showed moderate weight reduction, though these differences were not significant (*p* = 0.148 and *p* = 0.225, respectively).

On the 8th postoperative day, the Ileus + Bioglue2 subgroup experienced a smaller reduction in body weight compared to the Ileus2 subgroup (*p* < 0.001). However, compared to the Control2 and Bioglue2 subgroups, the weight loss in the ileus + Bioglue2 subgroup was greater, though not significant (*p* = 0.134 and *p* = 0.225, respectively).

**Figure 2:**
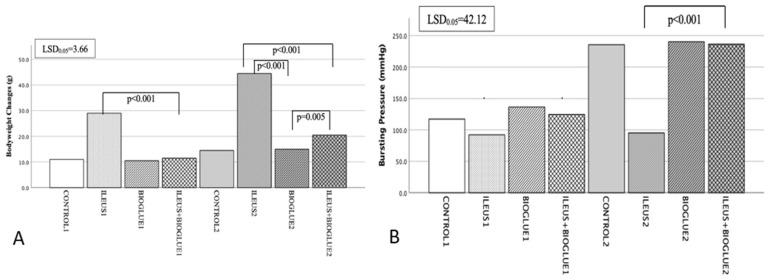
Body weight and bursting pressure changes: (**A**) body weight alteration per group and day; (**B**) bursting pressure (mmHg) per group and day.

When comparing the ileus + Bioglue group between postoperative days 4 and 8, a significantly greater reduction in body weight was observed on day 8 (*p* < 0.001), indicating a progressive decline over time (Figure 2A).

### 3.2. Bursting Pressure

The bursting pressure results among the groups are presented in Table S2, Supplementary Materials. Bursting pressure was zero in the rats in which anastomotic dehiscence was noted.

On the 4th postoperative day, the mean bursting pressure in the ileus + Bioglue1 subgroup was higher compared to the ileus1 subgroup (*p* = 0.130) but did not reach statistical significance. It was also higher than in the control1 and Bioglue1 subgroups, but the differences were not significant (*p* = 0.289 and *p* = 0.316, respectively).

On the 8th postoperative day, the ileus + Bioglue2 subgroup demonstrated a significantly higher mean bursting pressure compared to the ileus2 subgroup (*p* < 0.001), confirming the positive effect of Bioglue on anastomotic strength under ileus conditions. While bursting pressure was also higher in the ileus + Bioglue2 subgroup compared to the control2 and Bioglue2 subgroups, these differences were significant (*p* = 0.067 and *p* = 0.115, respectively).

Between the 4th and 8th postoperative days, the ileus + Bioglue group showed a significant increase in bursting pressure (*p* < 0.001), reflecting the improved mechanical strength of the anastomosis over time (Figure 2Β).

### 3.3. Anastomotic Dehiscence

On the 4th and 8th postoperative days of sacrifice, according to the protocol of the experiment, the removed part of the colon was examined macroscopically to determine whether the anastomosis was intact. The frequency of rupture of the anastomosis was evaluated on both 4th and 8th postoperative days (Table S3, Supplementary Materials).

On the 4th postoperative day, the incidence of anastomotic dehiscence in the ileus + Bioglue1 subgroup was lower than in the ileus1 subgroup (one rat—10% vs. two rats—20%), but this difference was not significant (*p* = 0.395). Compared to the control1 and Bioglue1 subgroups (0% in both), the ileus + Bioglue1 subgroup had a higher frequency of dehiscence, though this was not significant (*p* = 0.500 for both comparisons).

On the 8th postoperative day, three rats (30%) in the ileus2 group and one rat (10%) in the ileus + Bioglue2 group experienced dehiscence. Therefore, the ileus + Bioglue2 subgroup had a lower incidence of dehiscence compared to the ileus2 subgroup, but this difference was not significant (*p* = 0.248). No dehiscence was observed in the control2 or Bioglue2 subgroups (*p* = 0.500 for both comparisons with ileus + Bioglue2).

**Figure 3:**
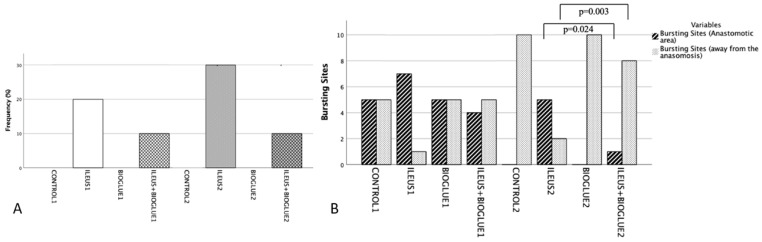
Anastomotic dehiscence: (**A**) frequency of anastomotic rupture per group; (**B**) frequency of rupture in the anastomotic area or away from it.

No significant difference in anastomotic dehiscence was observed within the ileus + Bioglue group between postoperative days 4 and 8 (10% on both days, *p* = 1.000) (Figure 3A).

### 3.4. Rupture Site

The rupture site was evaluated during bursting pressure testing, with ruptures categorized as occurring either at the anastomotic site or away from the anastomosis. The effects of the subgroups and the day of sacrifice in the rupture site are presented in Table S4, Supplementary Materials.

On the 4th postoperative day, the rupture at the anastomotic site was observed in the ileus + Bioglue1 subgroup at a lower frequency compared to the ileus1 subgroup (20% vs. 30%), but this difference was not significant (*p* = 0.081). Compared to the control1 and Bioglue1 subgroups, where no ruptures were observed (0%), the ileus + Bioglue1 subgroup had a higher rupture frequency at the anastomotic site, though these differences were also not significant (*p* = 0.344 and *p* = 0.272, respectively). Ruptures away from the anastomosis were not noted in the ileus + Bioglue1 subgroup on the 4th day.

On the 8th postoperative day, rupture at the anastomotic site occurred significantly less frequently in the ileus + Bioglue2 subgroup compared to the ileus2 subgroup (20% vs. 40%; *p* = 0.024), indicating the enhanced mechanical strength of the anastomosis. However, ruptures away from the anastomosis were more frequent in the ileus + Bioglue2 subgroup compared to the ileus2 subgroup (30% vs. 10%; *p* = 0.003), suggesting a shift in the rupture site due to improved anastomotic integrity. No ruptures were observed in the control2 and bioglue2 subgroups at either site.

The frequency of rupture at the anastomotic site in the ileus + Bioglue group decreased from 20% on the 4th postoperative day to 10% on the 8th day, but this difference was not significant (*p* = 0.564). Conversely, ruptures away from the anastomosis increased significantly between days 4 and 8 (0% vs. 30%; *p* = 0.008), indicating that as the strength of the anastomosis improved over time, ruptures shifted to less stable bowel segments (Figure 3Β).

### 3.5. Adhesion Formation

The evaluation of adhesions was performed during the macroscopic examination of the anastomosis, according to the van der Hamm scale [52]. The frequency of occurrence of the different adhesion grades per group and day is described in Table S5, Supplementary Materials.

On the 4th postoperative day, the adhesion scores in the ileus + Bioglue1 subgroup were significantly higher compared to the control1 and ileus1 subgroups (*p* < 0.001 for both comparisons). However, the adhesion scores in the ileus + Bioglue1 subgroup were comparable to those in the Bioglue1 subgroup (*p* = 0.272).

**Figure 4:**
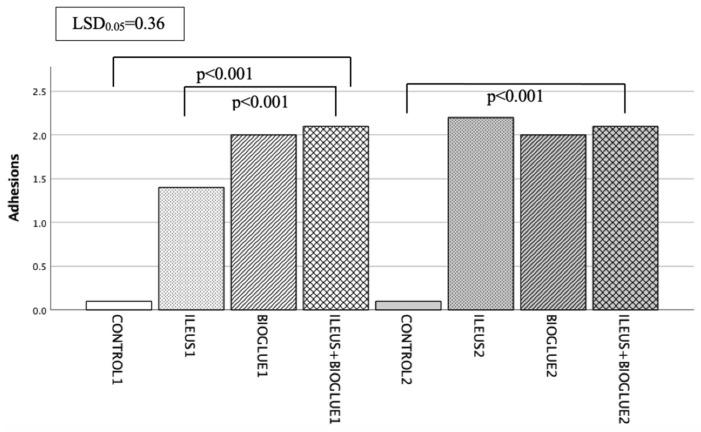
Adhesion formation per group and day.

On the 8th postoperative day, the ileus + Bioglue2 subgroup showed more adhesions compared to the ileus2 subgroup, though the difference was not significant (*p* = 0.235). The adhesion scores were significantly higher in the ileus + Bioglue2 subgroup compared to the control2 subgroup (*p* < 0.001) but not significantly different from the Bioglue2 subgroup (*p* = 0.288) Adhesion formation in the ileus + Bioglue group increased significantly between days 4 and 8 (*p* < 0.001), indicating progressive fibrin deposition over time (Figure 4).

### 3.6. Histological Assessment

The segments from the anastomotic region were microscopically assessed after haematoxylin and eosin staining. The classification was semiquantitative/categorical and was based on the Erlich–Hunt scale, as amended by Philips. The analysis was performed in a blind fashion by an experienced pathologist. In each group, leukocytosis, neovascularization, fibroblasts, and neo-collagen formation were evaluated.

#### 3.6.1. Leukocytosis

Τhe results of the effect of subgroups and day of sacrifice on leukocytosis are presented in Table S6, Supplementary Materials.

**Figure 5:**
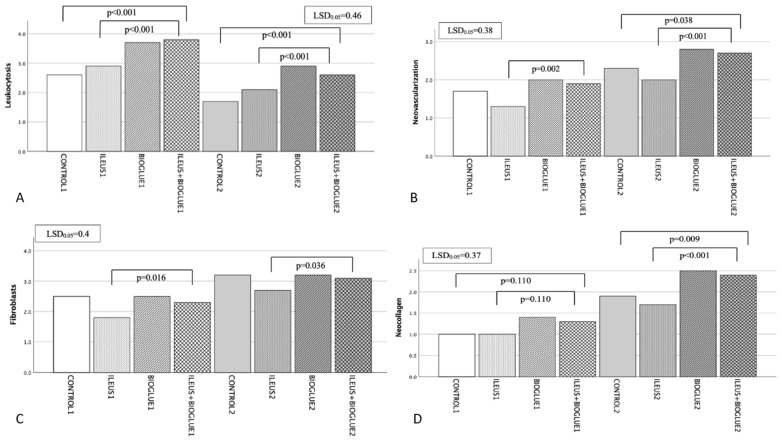
Histopathology: (**A**) leukocytosis per group and day; (**B**) neovascularization per group and day; (**C**) fibroblasts per group and day; (**D**) neocollagen per group and day.

On the 4th postoperative day, the mean leukocyte count in the ileus + Bioglue1 subgroup was significantly higher than in the ileus1 and control1 subgroups (*p* < 0.001 for both). Based on hematoxylin and eosin staining, on the 8th postoperative day, the ileus + Bioglue2 subgroup had a significantly higher leukocyte count, predominantly composed of neutrophils, than the ileus2 and control2 subgroups (*p* = 0.034 and *p* < 0.001, respectively). Leukocytosis decreased significantly in the ileus + Bioglue group between days 4 and 8 (*p* < 0.001), indicating the resolution of the inflammatory response (Figure 5A).

#### 3.6.2. Neovascularization

The results of the effect of subgroups and day of sacrifice on neovascularization are presented in Table S7, Supplementary Materials.

On the 4th postoperative day, the ileus + Bioglue1 subgroup exhibited significantly higher neovascularization compared to the ileus1 subgroup (*p* = 0.002), though not significantly different from the control1 or Bioglue1 subgroups (*p* = 0.161 and *p* = 0.089, respectively). On the 8th postoperative day, neovascularization in the ileus + Bioglue2 subgroup was significantly higher than in the ileus2 and control2 subgroups (*p* < 0.001 and *p* = 0.038, respectively). Neovascularization increased significantly in the ileus + Bioglue group between days 4 and 8 (*p* < 0.001), indicating enhanced vascular regeneration (Figure 5B).

#### 3.6.3. Fibroblast Activity

The results of the effect of subgroups and day of sacrifice on fibroblasts count are presented in Table S8, Supplementary Materials.

On both the 4th and 8th postoperative days, fibroblast activity was significantly higher in the ileus + Bioglue subgroup compared to the ileus subgroup (*p* = 0.016 for both days). Comparisons with the control and Bioglue subgroups showed no significant differences on the 4th day (*p* > 0.05) but revealed increased activity on the 8th day (*p* = 0.048 for control2 and *p* = 0.045 for Bioglue2)

Between days 4 and 8, fibroblast activity increased significantly (*p* < 0.001) in the ILEUS+BIOGLUE group, reflecting progressive connective tissue repair (Figure 5C).

#### 3.6.4. Neocollagen Production

The results of the effect of subgroups and day of sacrifice on neocollagen production are presented in Table S9, Supplementary Materials.

On the 4th postoperative day, the ileus + Bioglue1 subgroup showed significantly higher collagen deposition compared to the ileus1 subgroup (*p* = 0.024), indicating that Bioglue promotes early collagen synthesis under obstructive ileus conditions. Collagen production in the ileus + Bioglue1 subgroup was also moderately higher than in the control1 and Bioglue1 subgroups, but these differences were not significant (*p* = 0.081 and *p* = 0.112, respectively).

On the 8th postoperative day, the ileus + Bioglue2 subgroup demonstrated significantly increased collagen deposition compared to the ileus2 subgroup (*p* < 0.001), reflecting a sustained positive effect of Bioglue on anastomotic healing. Comparisons with the control2 and Bioglue2 subgroups revealed moderately higher collagen deposition in the ileus + Bioglue2 subgroup, though the differences did not reach statistical significance (*p* = 0.089 and *p* = 0.127, respectively).

Collagen deposition in the ileus + Bioglue group increased significantly between the 4th and 8th postoperative days (*p* < 0.001), indicating progressive and enhanced collagen production over time. This finding highlights the role of Bioglue in accelerating and sustaining the tissue repair process under conditions of obstructive ileus (Figure 5D).

#### 3.6.5. Hydroxyproline Levels

The results of the effect of subgroups and day of sacrifice on the concentration of hydroxyproline are presented in Table S10, Supplementary Materials.

**Figure 6:**
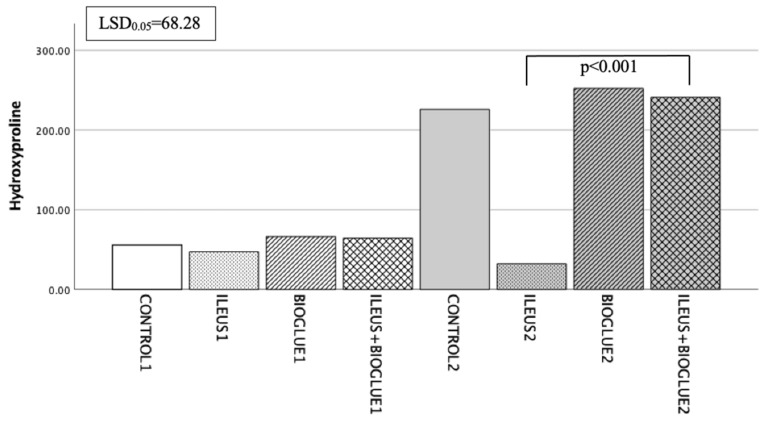
Hydroxyproline levels per group and day.

On the 4th postoperative day, the concentration of hydroxyproline in the ileus + Bioglue1 subgroup was higher compared to the control1 and ileus1 subgroups, but this difference was not significant (*p* = 0.778 and *p* = 0.619, respectively). On the 8th postoperative day, a significantly higher hydroxyproline concentration was specifically observed in the ileus + Bioglue2 subgroup compared to the ileus2 subgroup (*p* < 0.001). Comparing hydroxyproline on the 4th and 8th days within the ileus + Bioglue group revealed a significant increase from the 4th to the 8th day (ileus + Bioglue1 vs. ileus + Bioglue2: *p* < 0.001). Between days 4 and 8, the hydroxyproline levels in the ileus + Bioglue group increased significantly (*p* < 0.001), indicating accelerated collagen deposition (Figure 6).

#### 3.6.6. Type I Collagenase

The results of the effect of subgroups and day of sacrifice on the concentration of collagenase are presented in Table S11, Supplementary Materials.

**Figure 7:**
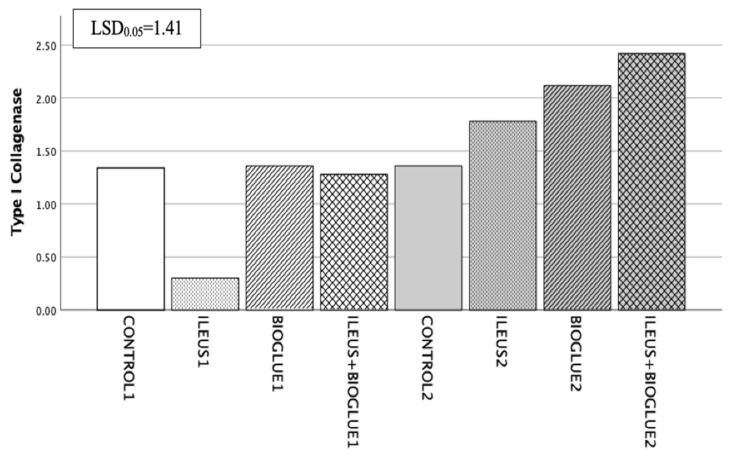
Type I collagenase per group and day.

On the 4th postoperative day, a higher collagenase concentration was observed in the ileus + Bioglue1 subgroup compared to the ileus1 subgroup, but this difference was not significant (*p* = 0.183). On the 8th postoperative day, the ileus + Bioglue2 subgroup had the highest concentration of collagenase among the subgroups ileus2 and Bioglue2, but this difference was not significant (*p* = 0.384 and *p* = 0.686, respectively). The collagenase concentration was increased from the 4th to the 8th postoperative day in the ileus + Bioglue group, but not significantly (ileus + Bioglue1 vs. ileus + Bioglue2: *p* = 0.123). Between days 4 and 8, the collagenase levels in the ileus + Bioglue group showed no statistically significant change (*p* = 0.123) (Figure 7).

## 4. Discussion

Anastomotic healing is a dynamic process and consists of a well-characterized series of events, which can be summarized into the following stages: coagulation, inflammation, proliferation, and remodeling [52]. In the beginning, hemostasis and preliminary spanning of the defect between colonic edges takes place, creating a hemostatic clot, which is infiltrated by migrating immune cells, especially leukocytes, to form the inflammatory infiltrate. This phase is characterized by a shift from pro- to anti-inflammatory signaling, in order to restrict endogenous inflammatory response [53]. During the proliferation stage, fibroblasts are the dominant cell type present in healing bowel tissue, aiming at collagen deposition, mainly of type III collagen, in the wound bed. This phase is also characterized by neoangiogenesis and the re-epithelialization of the bowel’s mucosal surface [54]. Finally, during the remodeling stage, collagen-degrading enzymes, called matrix metalloproteinases (MMPs), work to remove type III collagen from granulation tissue and form collagen type I [53].

Despite technological progress in colorectal surgery, impaired anastomotic healing may occur in up to 20% of cases, leading to anastomotic leakage, which is defined as the communication between the intraluminal and extraluminal space [53]. Anastomotic leakage has been associated with high morbidity and short-term mortality rates of up to 39%, increased length of hospital stay, and higher healthcare costs [55]. Numerous several patient-specific, surgical, and surgeon-related factors have been implicated in the disruption of anastomotic healing after colon and rectal surgery [56]. Many studies have focused on the effect of postoperative ileus on colonic anastomosis [57,58]. However, the role of preoperative ileus on colonic anastomotic integrity, such as in emergency cases of obstructive neoplasms or masses, has been scarcely described.

It is well-known that 10–28% of patients with colorectal cancer present with obstructive ileus symptoms as the first manifestation of the disease, while 10% of them will need urgent surgical treatment [59]. Obstructive colonic malignancies are a risk factor for poor postoperative prognosis, since immediate postoperative mortality ranges from 15 to 30% versus 1 to 5% in elective cases. Poor postoperative outcomes after emergency surgery for obstructive colon malignancy are multifactorial and are related to the consequences of obstruction, to the advanced stage of the tumor, and to suboptimal resection under emergency circumstances [60]. Based on the expertise and clinical experience of a group of national clinical specialists, the EUPEMEN Protocol has been proposed, in an effort to minimize surgical stress, improve postoperative outcomes and enhance the recovery of patients presenting with bowel obstruction [61].

Obstruction is usually located in the left colon, as its diameter is smaller and its contents denser [62]. Ileus causes local and systemic disorders, which inhibit the healing process of anastomosis and lead to an increased frequency of anastomotic leakage. More specifically, ileus impairs the anastomosis blood supply, which is crucial because of the limited, even in normal conditions, capillary density of the large intestine [63]. In addition, ileus causes increased intraluminal pressure, which leads to intestinal ischemia, and as ischemia prolongs, mucosa ulcerates due to epithelial cell destruction. Over time, lesions extend to the muscularis propria and involve the full thickness of the intestinal wall, becoming irreversible, which can lead to bowel perforation [64,65]. Intestinal ischemia-reperfusion (IR) is a common phenomenon after obstructive ileus resolves and is associated with high morbidity and mortality [66]. Ischemic lesions, the reduction in intestinal wall capillaries, and the secretion of vasoconstriction substances result in disruption of the anastomotic blood supply and inhibit anastomotic healing [67].

In recent years, scientific interest has been focused on the study of systemic and local anastomotic protection factors, aiming to enhance the healing process [68]. The most promising results have been observed with the use of the intraluminal latex implant, mechanical protection using a biofragmentable ring (BAR), the administration of growth factors (EGF, PDGF), and the application of bioadhesives (fibrin glue, fibrin sealant, Floseal) [69,70,71]. Various adhesive biological materials have been tested from time to time, aiming to enhance mechanical strength and to reduce the incidence of anastomotic dehiscence. These fibrous adhesives are biodegradable and biocompatible and have been used in numerous surgical procedures [72,73]. In particular, the literature contains studies investigating the therapeutic compounds used to promote anastomosis healing under obstructive ileus conditions in experimental models. These therapeutic strategies include intraoperative colonic lavage with povidone iodine (PI) [74] or NG-NitroL-Arginine Methyl Ester (L-NAME) [75], subcutaneous erythropoietin administration [76], intraperitoneal injection of iloprost [40], and local application of granulocyte macrophage-colony-stimulating factor [77].

Albumin/Glutaraldehyde (Bioglue^®^, CryoLife, Atlanta, GA, USA) is an FDA-approved tissue sealant used in various surgical procedures, including vascular, cardiac, and intra-abdominal surgeries [78]. The sealant syringe is easy to use and contains two components: purified bovine serum albumin and glutaraldehyde. Initially, it was mostly applied in cardiac surgery as a complementary tool for adhesion, sealing, and tissue strengthening. Its characteristic is that it creates a hydrophobic mechanical barrier protecting the surrounding tissue, thus preventing the escape of fluid and air, and consequently, perianastomotic leakage. It has also been found to affect the healing of anastomosis by increasing angiogenesis and fibroblasts proliferation and thereby promoting collagen production [79,80,81]. This adhesive can be used as an additional procedure to the established method of surgical repair strengthening, especially for soft tissues, such as the cardiac, pulmonary, vascular, urogenital, and of gastrointestinal tissues [82,83]. The advantages of its use are as follows: (1) rapid hemostasis, (2) improved anastomoses tightness, (3) reduced surgery time, (4) minimized blood loss, especially after cardiac surgery, and (5) reduced number of re-operations due to hemorrhaging [84,85]. It consists of a combination of two components, including a 10% glutaraldehyde solution and a 45% purified bovine albumin solution, which are kept separately until its application. Bovine serum albumin is derived from bovine herds in North America, which are free from bovine spongiform encephalopathy. It is converted to purified serum after cooling, chromatography, and gamma radiation. Its mechanism of action is based on the chemical reaction between aldehydes and amines. The amines for this reaction are administered by the amino-groups of the albumin lysine and by the extracellular tissue proteins. Glutaraldehyde is a dual-acting aldehyde that acts as a bridge, joining the lysine (albumin) groups with the extracellular proteins and the cell surface [84,86,87]. The result of this action is the formation of a permanent covalent bond between the tissues and the adhesive. This reaction increases the mechanical strength of the tissues regardless of the patient’s coagulation state. The high concentration of albumin present in albumin/glutaraldehyde (Bioglue), in combination with the rapid reaction between aldehydes and amines, allows this adhesive to stabilize in 20–30 sec and ensure maximum complex strength within 2–3 min [84,85]. The purpose of this experimental study was to investigate the action of the local application of the albumin/glutaraldehyde (Bioglue^®^) glue on the healing of colon anastomoses in rats undergoing colonic anastomosis under conditions of obstructive ileus.

Patients with colorectal cancer are generally characterized by decreased body weight and impaired preoperative malnutrition status, which have been associated with greater postoperative morbidity after resection [88]. In addition, malnutrition negatively affects the anastomotic healing process by impairing collagen synthesis and fibroblast proliferation, thereby reducing anastomotic mechanical strength [89]. In our study, on the 4th and 8th postoperative days, the use of the glue under obstructive ileus conditions led to a smaller reduction in average body weight compared to the rats with ileus.

According to Vakalopoulos et al. [17], the reported incidence of anastomotic leakage ranges from 5% to 25%, with an associated mortality rate of up to 32%. The percentage of anastomotic leakage is the most indicative marker reflecting a sufficient healing process [17]. Regarding anastomotic dehiscence, our results show that the administration of Bioglue adhesive had a positive effect on the waterproofing of the anastomosis, as no leakage was observed in the rats in which glue was applied. The effect of the adhesive was also protective in obstructive ileus conditions, as the incidence of leakage was lower in the rats in which glue was applied after the ileus compared to those with ileus, but this comparison was not significant. In addition, the use of the glue under obstructive ileus conditions resulted in a lower incidence of rupture in anastomosis compared to ileus alone, and this difference was significant. A similar beneficial role in decreasing the anastomotic leakage after the resection of colonic obstruction seems to be played by AZD3342, a selective MMP-8, MMP-9, and MMP-12 inhibitor [90]. A 2022 systematic review and meta-analysis confirmed the above in humans, demonstrating a leakage reduction in patients in whom intestinal anastomosis was coated by fibrin adhesives or collagen-based laminar biomaterials [19]. The literature also contains reports of therapeutics offering enhanced anastomosis protection under artificially obstructive conditions in rats, such as intraperitoneal iloprost and tacrolimus [91].

A successful anastomotic healing process is defined by the ability of bowel anastomosis to withstand tensile forces and can be assessed by measuring bursting pressure, which is associated with anastomosis mechanical strength [92]. In our experiment, on the 4th postoperative day, the mean bursting pressure in the ileus subgroup that received albumin/glutaraldehyde was higher compared to the untreated ileus subgroup, although the difference was not statistically significant. However, this difference was significant on the 8th postoperative day. The observation that the mean bursting pressure was significantly higher on day 8 compared to day 4 when glue was used, even after ileus, whereas no statistical significance was found in the rats that experienced ileus, demonstrates the positive effect of adhesive on the healing of anastomosis in both normal and obstructive ileus conditions. On the contrary, a study in rats that examined the use of fibrin glue only under low-risk conditions did not find any difference regarding bursting pressure between the control group and the group in which glue was applied in the anastomosis [93].

During the early post-operative period, collagenolysis decreases the collagen levels at the site of anastomosis up to 40%, leading to an increased rate of anastomotic dehiscence at the anastomotic site during this period. In contrary, with the progressive increase in collagen formation from day 5, the mechanical strength of anastomosis increases, and ruptures usually occur away from the anastomosis in focal necrosis sites [94]. In our study, the frequency of rupture away from anastomosis during the 4th postoperative day did not differ significantly among the groups because in these anastomoses the healing process was at an early stage. On the 8th postoperative day, the lowest rupture frequency away from anastomosis was observed in the rats of the ileus group compared to all other subgroups, and this difference was significant. In addition, the rats treated with glue application after ileus had a higher frequency of rupture away from anastomosis than the ileus subgroup, and this difference was significant. This means that the application of the adhesive has the effect of increasing the strength of the anastomosis between the 4th and 8th day, both in the adhesive group and in the ileus and adhesive groups, presenting a positive effect on the healing process of anastomosis, both in normal and obstructive ileus conditions.

Fibrinous adhesions are formed between intestinal loops, other abdominal viscera, and the peritoneum in the early period after surgery, particularly in intestinal ischemia, inflammation, or abdominal trauma [95]. Adhesions have a protective effect on the anastomotic healing process because of their ability to cover small defects of the suture line and to increase vascularity in the hypoperfused sites of the anastomosis [96]. The grade of adhesion formation is affected by the severity of intestinal trauma, fibroblast activity, the action of metalloproteinases, and the efficacy of the fibrinolytic mechanisms [35]. In our experiment, on the 4th postoperative day, the ileus and glue subgroups showed significant more adhesions compared to the ileus subgroup, while on the 8th postoperative day, the ileus subgroup showed more adhesions compared to the ileus + adhesive subgroup, with no significant difference. In addition, adherence testing between the control and Bioglue groups revealed significantly more severe adhesions in the glue group on both the 4th and 8th postoperative days. The effect of the adhesive, therefore, resulted in an increased number of adhesions on both days of sacrifice. These results agree with the findings of van der Ham et al., who concluded that fibrin glue increases the adhesion formation of colonic anastomosis [45]. In contrary, Ceran et al. concluded that postoperative intraperitoneal administration of ghrelin after colonic anastomosis leads to lower adhesion formation rates and more loose adhesions [42].

Immune system cells, and mainly polymorphonuclear cells, have a crucial role in the regulation of the anastomosis healing, through the secretion of molecular signals [97]. In our study, the application of adhesive resulted in an increase in leukocytes count in the glue and ileus and the glue groups compared to the control and ileus groups on both days of sacrifice, while the mean leukocyte counts on the 8th postoperative day were significantly reduced in all groups compared to the 4th day, as a normal development in the process of healing the bowel anastomosis. Similarly, Vakalopoulos et al. reported that Bioglue induced the most intense short-term inflammatory response, due to direct toxic effect on bowel and due to its initial degradation into toxic by-products [98]. Ekici et al., investigating the role of Bioglue on high-risk left colon anastomosis after the intraperitoneal injections of 5-fluorouracil (5-FU), found no difference in inflammatory cell infiltration after the application of tissue glue [34].

Furthermore, on the 4th postoperative day, the ileus led to the least neovascularization compared to all other subgroups, and this difference was significant. Similarly, on the 8th postoperative day, neovascularization was reduced in the subgroup that experienced obstructive ileus compared to all other subgroups, but this difference was significant in the subgroups in which glue was applied, both under and in the absence of ileus conditions.

Neoangiogenesis is an important factor for sufficient anastomotic healing through vascularity increase and new tissue formation [99]. We noticed that the local application of the adhesive increased neovascularization on both days of sacrifice, both in normal conditions and in the obstructive ileus conditions, promoting neoangiogenesis. Similar results have emerged from a very recent study, in which fibrin glue was found to increase angiogenesis in rats that underwent enterotomy and ileoileal anastomosis [96].

Fibroblasts are the main source of the main constituents of the connective tissue, such as collagen, proteoglycans, and elastins, and their migration to the anastomotic site increases markedly on the second postoperative day, returning to the normal level afterwards [100]. Therefore, concerning the effect of Bioglue on fibroblasts in both normal and obstructive ileus conditions, an increase in their number was observed in the rats in which glue was applied, both in the absence and under ileus conditions compared to those that presented ileus, a difference that was significant on both the 4th and 8th postoperative days. Similarly, a recent study showed that TISSEEL, another fibrin sealant, increases the fibroblast concentration both in euglycemic and diabetic rats in which colorectal anastomosis was performed [101]. In addition, Ekici and colleagues proved that Bioglue reversed the negative effects of 5-FU on fibroblast proliferation and activity in colonic anastomosis [34].

In our study, on the 4th postoperative day, neocollagen production was higher in the rats in which with glue was use after ileus compared to the ileus subgroup, but this difference was not significant. On the 8th postoperative day, neocollagen production was found to be higher in the ileus + Bioglue subgroup compared to the ileus group, and this difference was significant. Therefore, the application of the adhesive, both under normal conditions and under obstructive ileus conditions, resulted in increased neocollagen formation in the rats in which glue was applied, even after ileus conditions, on both the 4th and 8th postoperative days, compared with the control and ileus subgroups. The beneficial role of glue, TISSEEL in this occasion, was demonstrated recently under both euglycemic and hyperglycemic conditions in rats [101]. Similarly, Yol et al. also concluded that the application of Bioglue on colonic anastomosis contributed to increased collagen formation and density [102].

Hydroxyproline levels are used for the biochemical evaluation of anastomotic healing, since low hydroxyproline levels are associated with poor anastomotic healing [103]. We found that, on the 4th postoperative day, the concentration of hydroxyproline after the use of glue under ileus conditions was increased compared to the control and ileus subgroups, but this difference was not significant. On the 8th postoperative day, the effect of the adhesive under ileus conditions was positive, leading to significant increased hydroxyproline levels in the ileus + Bioglue group compared to ileus group. These results show that the increase in collagen was not only qualitative but also quantitative, leading to increased anastomotic strength. Van de Ham et al. found similar results while examining the use of fibrin glue in rats under normal conditions [45].

Type I collagenase belongs to the metalloproteinases family and is the initiating enzyme responsible for mature collagen degradation from the submucosal layer, contributing to colonic anastomotic dehiscence. In this way, the collagenase concentration assay is indicative of the balance between collagenogenesis and collagenolysis [104]. In our study, on both sacrifice days, the ileus +Bioglue group exhibited a higher collagenase concentration compared to the ileus subgroup, but with no significant difference, with an increase on its levels noticed from the 4th to the 8th postoperative days.

Regarding the safety profile, our study showed that no deaths or other adverse effects were caused by the application of Bioglue on anastomotic line. However, Bioglue has been associated with toxic adverse effects in various tissue types, such as the phrenic nerve and cardiac conduction tissues. It has also been reported that the toxic effect increases after the contact of Bioglue with incisional tissue surfaces. Thus, application to the outer layer and vascular tissues is recommended [34]. In addition, concerns regarding its long-term biocompatibility remain, as glutaraldehyde-based adhesives have been associated with inflammatory responses and potential foreign body reactions in some experimental models [34]. Long-term results after glue application in aortic tissues revealed severe active inflammation and massive foreign body reaction [105]. In addition, Bioglue application in mouse colonic anastomosis has resulted in increased rates of mechanical ileus, as well as increased mortality rates, according to Slieker et al. [106].

Previous studies have explored the effects of Bioglue on anastomotic healing under normal conditions, as well as the impact of obstructive ileus on anastomotic integrity [35,36]. However, the present study is the first to evaluate the combined effect of Bioglue application in the setting of obstructive ileus, a condition known to impair anastomotic healing through ischemia, inflammation, and increased mechanical stress. Our findings demonstrate that Bioglue enhances key aspects of tissue repair—including collagen deposition, neovascularization, and fibroblast activity—even under the compromised conditions of ileus. This suggests that Bioglue may mitigate the detrimental effects of ileus on anastomotic healing, providing a potential strategy for reinforcing anastomoses in high-risk surgical scenarios. These insights extend previous research and highlight the relevance of Bioglue beyond elective cases, supporting its possible application in emergency colorectal surgery where obstructive ileus is a common challenge.

The role of Bioglue in anastomotic reinforcement must be considered in the context of other bioadhesives used in gastrointestinal surgery. Fibrin glue, despite its biocompatibility and ease of application, has limited mechanical strength and undergoes rapid degradation, reducing its ability to provide long-term structural support [107]. Additionally, its reliance on exogenous thrombin raises concerns about immune reactions and variable clot stability in patients with coagulopathies. Cyanoacrylate-based adhesives offer strong bonding capabilities but suffer from high tissue toxicity, leading to local inflammation, necrosis, and potential foreign body reactions, which restrict their use in gastrointestinal surgery [108]. A systematic review by Vakalopoulos et al. [17] extensively analyzed the use of tissue adhesives in gastrointestinal anastomoses, highlighting both their potential benefits and limitations. The review found that while fibrin glue has been widely studied, it does not consistently reduce anastomotic leakage rates in colorectal surgery. Additionally, cyanoacrylate glues, despite their strong adhesive properties, were noted to cause foreign body reactions and fibrosis, limiting their clinical applicability. The review further emphasized that the variability in study methodologies and adhesive formulations makes direct comparisons challenging, and no single adhesive has yet emerged as the gold standard for colorectal anastomotic reinforcement.

There are some limitations to be considered in this study. First, this study was conducted on experimental animals, and a large number of animal studies are needed to confirm the utility of the data in humans. As a result, while the results provide valuable insights into the effects of Bioglue on colonic anastomotic healing under obstructive ileus conditions, caution is needed when interpreting these findings, as they cannot be directly applied to clinical practice. Furthermore, the relatively small sample size of this study limits the statistical power of our findings. Although significant trends were observed, a larger sample would be necessary to draw more definitive conclusions. In addition, our study does not provide long-term follow-up data on the effect of Bioglue on colonic tissues, which are also scarce in literature. Bridging the gap between experimental findings and clinical application requires further research. In this way, further large-scale experimental studies and clinical trials are required in order to assess the effects of Bioglue on anastomotic healing after direct application to the colon, as well as the safety profile and long-term outcomes. In addition, studies with an increased number of animals could enhance the reliability of the results and allow for more robust statistical analyses and applicability of findings in surgical settings. Additionally, comparative studies with existing clinical interventions would provide a better context for its potential role in surgical practice.

The findings of this study suggest that Bioglue may have potential applications in colorectal surgery, particularly in cases where anastomotic integrity is compromised due to ischemia, infection, or high-risk surgical conditions. Given the limited clinical evidence available, further research is necessary to evaluate its efficacy and safety in human colorectal anastomoses. Future studies should focus on large-animal models, such as porcine models, which closely resemble human colorectal physiology, to better assess Bioglue’s mechanical properties and biocompatibility. Additionally, well-designed clinical trials are essential to determine whether Bioglue can reduce anastomotic leakage rates, improve healing outcomes, and minimize postoperative complications in high-risk surgical patients. Expanding research to alternative surgical applications, including minimally invasive and robotic-assisted colorectal procedures, may further clarify its role in modern surgical practice.

Statistical power is a fundamental aspect of study reliability, as it determines the likelihood of detecting true effects. In this study, while the trends suggest a potential benefit of Bioglue in anastomotic healing, the small sample size limits the ability to draw definitive conclusions. The relatively limited number of animals per group increases the risk of Type II errors, potentially obscuring meaningful differences. Although ANOVA and Fisher’s exact tests were employed for statistical analysis, the limited sample size reduces the robustness of these comparisons. To partially address this, *p*-values at a slightly higher threshold (*p* < 0.10) were reported for certain analyses; however, this does not substitute for the need for larger-scale studies.

Enhancing statistical power in future research requires a larger sample size to improve effect estimate precision and reduce both Type I and Type II errors. The application of advanced statistical methodologies, such as mixed-effects modeling, could provide better control for individual variability and increase analytical robustness. Furthermore, meta-analyses of multiple experimental studies could offer a more comprehensive assessment of Bioglue’s impact on anastomotic healing. Given these limitations, the findings of this study should be interpreted as preliminary, underscoring the need for further large-scale experimental and clinical investigations to confirm their validity and clinical applicability.

In conclusion, anastomotic leakage after colonic resection is a serious complication, associated with increased postoperative morbidity and mortality rates. The rate of anastomotic dehiscence is also increased under obstructive ileus conditions. Our study proved that the local application of albumin/glutaraldehyde (Bioglue) anastomosis healing both on the 4th and on the 8th postoperative days did no lead to anastomotic leakage and increased mechanical strength. Specifically, the application of Bioglue increased inflammatory response, neoangiogenesis, fibroblasts proliferation, and neocollagen production under obstructive ileus conditions. Therefore, local application of albumin/glutaraldehyde (Bioglue) on colonic anastomosis seems to have a protective and reinforcing effect on the healing process of colonic anastomoses, in conditions of obstructive ileus, both on the 4th and on the 8th postoperative days.

## 5. Conclusions

Anastomotic dehiscence remains a major complication in colorectal surgery, particularly under obstructive ileus conditions. Our study demonstrates that the application of albumin/glutaraldehyde (Bioglue) enhances colonic anastomotic healing by improving mechanical strength, promoting fibroblast activity, neovascularization, and collagen deposition. While a trend toward lower anastomotic dehiscence was observed, statistical significance was not reached. These findings suggest that Bioglue may mitigate the adverse effects of ileus on anastomotic integrity, reducing the risk of dehiscence. However, its clinical applicability in colorectal surgery remains uncertain due to the lack of large-scale clinical trials evaluating its long-term biocompatibility and safety. While it may be beneficial for high-risk anastomoses, particularly in cases with ischemia, peritonitis, or intra-abdominal sepsis, further research is required to assess its degradation profile, potential inflammatory response, and effects on tissue remodeling. This study has some limitations, including a modest sample size, the exclusive use of male rats, which prevents the assessment of sex-based differences in anastomotic healing, and the absence of immunohistochemical analysis to further investigate inflammation, fibrosis, and angiogenesis. Future studies should include both male and female subjects, incorporate large-animal models for better translational relevance, and compare Bioglue with other bioadhesives to determine its relative efficacy. Additionally, long-term safety evaluations and clinical trials are needed to establish whether Bioglue can enhance anastomotic integrity in colorectal surgery, particularly for high-risk patients.

## Data Availability

Data are available to any qualified researchers upon request to telonakos@gmail.gr.

## Supplementary Materials

The following supporting information can be downloaded at: https://www.mdpi.com/article/10.3390/jcm14072457/s1. Table S1: Body weight (gr) before the experiment and on the sacrifice day; Table S2: Bursting pressure (mmHg) on 4th and 8th postoperative day; Table S3: Frequency of the rupture of the anastomosis per group and day; Table S4: Rupture site per group and day; Table S5: Frequency of adhesion formation per group and day; Table S6: Leukocytosis; Table S7: Neovascularization; Table S8: Fibroblasts; Table S9: Neocollagen; Table S10: Hydroxyproline; Table S11: Type I Collagenase.
