# Supplementary material for: The Effect of Albumin/Glutaraldehyde Glue (Bioglue) on Colonic Anastomosis Under Intestinal Obstruction: An Experimental Study in Rats
Source: J Clin Med. 2025 Apr 3;14(7):2457. doi: 10.3390/jcm14072457 (PMC11989762; doi:10.3390/jcm14072457)
Supplement: Supplementary file 1 [file jcm-14-02457-s001.zip › jcm-3413442-supplementary.pdf]

Table S1. Body weight (gr) before the experiment and on the sacrifice day.

Group	n	Mean	95% C.I.	Median	SD
**4th postoperative day**					
**CONTROL**					
**Before experiment**	10	278.0	242.9-296.2	282.5	17.51
**Before sacrifice**	10	267.0	268.8-287.1	272.5	16.70
**ILEUS**					
**Before experiment**	10	284.5	261.2-298.8	285.0	11.65
**Before sacrifice**	10	255.5	275.3-293.6	262.5	14.23
**BIOGLUE**					
**Before experiment**	10	263.5	244.0-282.9	265.0	9.73
**Before sacrifice**	10	253.0	254.3-272.6	252.5	9.78
**ILEUS+BIOGLUE**					
**Before experiment**	10	266.5	219.1-299.8	272.5	23.69
**Before sacrifice**	10	255.0	257.3-275.6	260.0	23.57
**8th postoperative day**					
**CONTROL**					
**Before experiment**	10	278.0	243.6-297.3	275.0	17.19
**Before sacrifice**	10	263.5	268.8-287.1	265.0	18.72
**ILEUS**					
**Before experiment**	10	281.5	260.9-298.0	282.5	10.29
**Before sacrifice**	10	237.0	272.3-290.6	230.0	11.11
**BIOGLUE**					
**Before experiment**	10	260.5	244.5-276.4	262.5	7.98
**Before sacrifice**	10	245.5	251.3-269.6	247.5	12.35
**ILEUS+BIOGLUE**					
**Before experiment**	10	271.0	249.5-292.5	270.0	10.75
**Before sacrifice**	10	250.5	261.8-280.1	250.0	10.39

n: sample size; SD: Standard Deviation; C.I.: Confidence Interval

Table S2. Bursting pressure (mmHg) on 4th and 8th postoperative day.

Group	n	Minimum	Maximum	Mean	SD	95% C.I.	Median
**CONTROL**							
**4th postoperative**	10	102	146	117.3	14.8	86.54-148.05	113

**day (CONTROL1)**							
**8th postoperative day (CONTROL2)**	10	192	268	235.6	24.4	204.84-266.35	240
**ILEUS**							
**4th postoperative day (ILEUS1)**	10	102	126	92.4	49.5	61.64-123.15	110
**8th postoperative day (ILEUS2)**	10	106	156	95.2	67.1	64.44-125.95	127
**BIOGLUE**							
**4th postoperative day (BIOGLUE1)**	10	112	156	136.6	13.5	105.84-167.35	137
**8th postoperative day (BIOGLUE2)**	10	222	265	240.3	13.4	209.54-271.05	242
**ILEUS+BIOGLUE**							
**4th postoperative day (ILEUS+BIOGLUE1)**	10	108	178	124.8	51.5	94.04-155.55	125
**8th postoperative day (ILEUS+BIOGLUE2)**	10	240	280	236.4	84.1	206.61-266.18	264

n: sample size; SD: Standard Deviation; C.I.: Confidence Interval

Table S3. Frequency of the rupture of the anastomosis per group and day.

**Group**	**n**	**Rupture**	**%**
**4th postoperative day**			

**CONTROL**	10	0	0
**ILEUS**	10	2	20
**BIOGLUE**	10	0	0
**ILEUS+BIOGLUE**	10	1	10
**8th postoperative day**			
**CONTROL**	10	0	0
**ILEUS**	10	3	30
**BIOGLUE**	10	0	0
**ILEUS+BIOGLUE**	10	1	10

n: sample size

Table S4. Rupture site per group and day.

Group	Rupture in the anastomotic area	Rupture away from the anastomosis	Total
**4th postoperative day**			
**CONTROL1**	5 (50%)	5 (50%)	10
**ILEUS1**	7 (87.5%)	1 (12.5%)	8
**BIOGLUE1**	5 (50%)	5 (50%)	10
**ILEUS+BIOGLUE1**	4 (44.4%)	5 (55.5%)	9
**8th postoperative day**			
**CONTROL2**	0	10 (100%)	10
**ILEUS2**	5 (71.42%)	2 (28.58%)	7
**BIOGLUE2**	0	10 (100%)	10
**ILEUS+BIOGLUE2**	1 (11.11%)	8 (88.88%)	9

Table S5. Frequency of adhesion formation per group and day.

Group/Grade	0	1	2	3
**4th postoperative day**				
**CONTROL1**	9 (90%)	1 (10%) (10%)	0	0
**ILEUS1**	0	8 (80%)	0	2 (20%)
**BIOGLUE1**	0	0	10 (100%)	0
**ILEUS+BIOGLUE1**	0	0	9 (90%)	1 (10%)
**8th postoperative day**				
**CONTROL2**	9 (90%)	1 (10%)	0	0
**ILEUS2**	0	0	8 (80%)	2 (20%)
**BIOGLUE2**	0	0	10 (100%)	0
**ILEUS+BIOGLUE2**	0	0	9 (90%)	1 (10%)

Table S6. Leukocytosis.

Group	Mean	SD	n
**4th postoperative day**			
**CONTROL1**	2.6	0.52	10
**ILEUS1**	2.9	0.74	10
**BIOGLUE1**	3.7	0.48	10
**ILEUS+BIOGLUE1**	3.8	0.42	10
**8th postoperative day**			
**CONTROL2**	1.7	0.48	10
**ILEUS2**	2.1	0.32	10
**BIOGLUE2**	2.9	0.57	10
**ILEUS+BIOGLUE2**	2.6	0.52	10

n: sample size; SD: Standard Deviation

Table S7. Neovascularization.

Group	Mean	SD	n
**4th postoperative day**			
**CONTROL1**	1.7	0.67	10
**ILEUS1**	1.3	0.48	10
**BIOGLUE1**	2.0	0.40	10
**ILEUS+BIOGLUE1**	1.9	0.32	10
**8th postoperative day**			
**CONTROL2**	2.3	0.48	10
**ILEUS2**	2.0	0.36	10
**BIOGLUE2**	2.8	0.42	10
**ILEUS+BIOGLUE2**	2.7	0.48	10

n: sample size; SD: Standard Deviation

Table S8. Fibroblasts.

Group	Mean	SD	n
**4th postoperative day**			
**CONTROL1**	2.5	0.53	10
**ILEUS1**	1.8	0.42	10
**BIOGLUE1**	2.5	0.53	10
**ILEUS+BIOGLUE1**	2.3	0.48	10
**8th postoperative day**			
**CONTROL2**	3.2	0.42	10
**ILEUS2**	2.7	0.48	10
**BIOGLUE2**	3.2	0.42	10

**ILEUS+BIOGLUE2**	3.1	0.38	10

n: sample size; SD: Standard Deviation

Table S9. Neocollagen.

Group	Mean	SD	n
**4th postoperative day**			
**CONTROL1**	1.0	0.26	10
**ILEUS1**	1.0	0.26	10
**BIOGLUE1**	1.4	0.52	10
**ILEUS+BIOGLUE1**	1.3	0.48	10
**8th postoperative day**			
**CONTROL2**	31.9	0.32	10
**ILEUS2**	1.7	0.48	10
**BIOGLUE2**	2.5	0.53	10
**ILEUS+BIOGLUE2**	2.4	0.52	10

n: sample size; SD: Standard Deviation

Table S10. Hydroxyproline.

Group	Minimum	Maximum	Mean	SD	Median	n
**4th postoperative day**						
**CONTROL1**	23.11	116.41	54.66	28.89	48.12	10
**ILEUS1**	15.5	136.36	47.28	37.28	39.21	10
**BIOGLUE1**	19.68	186.25	66.23	43.89	46.51	10

**ILEUS+BIOGLUE1**	19.3	194.4	64.38	40.24	37.44	10
**8th postoperative day**						
**CONTROL2**	127.34	338.17	255.89	53.67	222.11	10
**ILEUS2**	10.79	134.25	32.27	32.62	17.9	10
**BIOGLUE2**	134.09	599.61	252.36	87.47	231.2	10
**ILEUS+BIOGLUE2**	186.4	310.74	241.13	72.2	210.96	10

n: sample size; SD: Standard Deviation

Table S11. Type I Collagenase.

Group	Minimum	Maximum	Mean	SD	Median	n
**4th postoperative day**						
**CONTROL1**	0.08	3.19	1.34	0.94	1.13	10
**ILEUS1**	0.11	0.94	0.31	0.24	0.24	10
**BIOGLUE1**	0.10	2.95	1.36	0.99	1.38	10
**ILEUS+BIOGLUE1**	0.12	3.16	1.28	1.08	1.08	10
**8th postoperative day**						
**CONTROL2**	0.01	4.74	1.36	1.46	0.92	10
**ILEUS2**	0.09	8.66	1.78	2.67	0.77	10
**BIOGLUE2**	0.16	7.7	2.12	2.61	0.88	10
**ILEUS+BIOGLUE2**	0.23	5.05	2.42	1.4	2.45	10

n: sample size; SD: Standard Deviation
